# Evaluating the Effect of Preventative Trimming on Distance from the Sole Surface to the Distal Phalanx Using Ultrasonography for Lameness Prevention in Pasture-Based Dairy Cows

**DOI:** 10.3390/vetsci10020077

**Published:** 2023-01-19

**Authors:** Chacha W. Werema, Linda J. Laven, Kristina R. Mueller, Richard A. Laven

**Affiliations:** 1 School of Veterinary Science, Massey University, Private Bag 11 222, Palmerston North 4442, New Zealand; 2 College of Veterinary Medicine and Biomedical Sciences, Sokoine University of Agriculture, Morogoro 67115, Tanzania

**Keywords:** claw trimming, claw sole thickness, ultrasonography, locomotion scoring, lameness, dairy cows

## Abstract

**Simple Summary:**

In pasture-based cattle, it is often suggested that claw trimming, a common lameness prevention practice in housed cows, results in thinner soles over the medium-to-long term. However, there is a lack of data on claw trimming in pasture-based cows. The aim of this study was to measure the effect of trimming on sole thickness in pasture-based cows and evaluate the effect of sole thickness on locomotion scores. Cows were randomly selected and allocated to ultrasound-trimming and ultrasound-non-trimming groups. In May 2018 (at drying off), the ultrasound-measured distance from the external claw sole surface to the pedal bone (distance to the pedal bone) was recorded. Then, trim-group cows were trimmed using the five-step Dutch method to trim the hindlimbs. These procedures were repeated during early lactation (October 2018) and toward the end of lactation (May 2019). The study found that trimming did not affect distance to the pedal bone and that there was no clear effect on the time to increased locomotion scores. The results of this study suggest that in pasture-based cattle, hoof trimming by a professional hoof trimmer using the Dutch method does not alter sole thickness over time.

**Abstract:**

One common management strategy used to reduce the risk of lameness is prophylactic claw trimming. However, in pasture-based cattle, there is a concern that the immediate reduction in sole thickness resulting from sole trimming will lead to medium-to-long-term reductions in sole thickness, which may increase the risk of lameness. Nevertheless, there is a lack of data on sole thickness and trimming in pasture-based cows. Therefore, the aim of this study was to evaluate the effect of trimming on sole thickness over the medium-to-long term, as estimated using the ultrasound-measured distance from the external claw sole surface to the distal phalanx (DDP) and of DDP on the interval between calving and increased locomotion scores. A total of 38 cows were randomly selected from a 940-cow spring calving dairy farm in the North Island of New Zealand; 18 were allocated to the ultrasound hoof-trimming group and 20 were allocated to the ultrasound non-trimming group. Starting in May 2018, at the end of the 2017/18 lactation, ultrasound measurements of DDP of the right hind hoof were made on all 38 cows, and the hindlimbs of the trimming group cows were trimmed by an experienced professional hoof trimmer using the five-step Dutch method. This was repeated in October 2018 (early lactation) and May 2019 (late lactation). After calving, the cows were locomotion scored fortnightly until the end of lactation using the 4-point (0–3) scale DairyNZ system. The effect of DDP on the interval between calving and the first locomotion scores ≥1 and ≥2 was assessed using Cox proportional hazards models, and the association between trimming and DDP was explored using linear mixed models. The results suggest that DDP has no effect on the time to locomotion scores ≥1 or ≥2, although the wide confidence intervals of the latter suggest that more data are needed before any definitive conclusions can be drawn. The study failed to find any clinically important impact of prophylactic trimming on DDP. This is likely related to the finding that cows with the highest DDP at the first trimming were identified by the hoof trimmer as those needing the most trimming. The results of this study thus suggest that if the Dutch five-step method is properly applied, it is unlikely to affect sole thickness over the short-to-medium term in pasture-based cattle.

## 1. Introduction

Claw horn disruption lesions (CHDL), primarily sole hemorrhages, ulcers, and white line disease, are the major challenge to decreasing lameness prevalence on dairy farms [1,2]. The principal cause of CHDL is contusions of the corium created by the distal phalanx [3]. The suspensory apparatus and the digital cushion are the primary structures preventing the distal phalanx from damaging the corium. The suspensory apparatus is a collection of collagenous fibrous connective tissue whose bundles are anchored in fibrous cartilage in the distal phalanx and transfer tensile forces to the horn capsule [4], while the digital cushion is a complex structure of fat and connective tissue that dampens and dissipates compressive forces in the heel and under the distal phalanx [4,5]. Thus, overstraining of the suspensory apparatus caused by housing, feeding, and management conditions resulting in the suboptimal function of the suspensory apparatus [3,4,6] and digital cushion [7,8,9] predisposes the corium to a high-pressure load, leading to contusions and, subsequently, CHDL.

Various factors have been shown to influence the function of the suspensory apparatus and digital cushion. For the suspensory apparatus, the key factor affecting its function appears to be the physiological changes associated with calving, which lead to an increase in laxity and a decrease in the strength of the suspensory apparatus [3,6,10,11]. For the digital cushion, it seems that the most important factors that influence its function are those that affect its thickness, such as stage of lactation, parity, body condition score, and inflammation within the hoof [3,7,8,12,13]. Studying such changes directly in a live animal is difficult, but ultrasonography can provide a guide to the distance between the distal phalanx and the sole surface [14,15,16], providing an indirect measurement of digital cushion thickness and the position of the distal phalanx within the horn capsule [13,17,18,19]. 

One commonly recommended measure to reduce the prevalence of CHDL is prophylactic trimming, which aims to restore normal claw conformation [20,21,22,23,24]. Most studies of prophylactic trimming have been undertaken with housed cattle and, as far as the authors are aware, only one peer-reviewed study has been published using data from cattle based permanently at pasture [25]. One concern associated with the use of prophylactic trimming is that it can result in thin soles, even when standard protocols are used, thus predisposing cattle to contusions due to a lack of protection of the sensitive corium [19,26,27,28,29]. The concern is not just that trimming can lead immediately to thin soles but that trimming can interact with abrasive environments to produce thin soles in the medium-to-long term [14,27]. This concern about the medium-to-long-term effects of trimming is prevalent in countries (such as New Zealand) that have pasture-based cattle that walk long distances from the pasture to the milking parlor at milking time, as it is assumed that walking long distances on farm tracks may have the same effect as being housed in a building with abrasive flooring [30]. However, there are no data on the medium-to-long-term impact of trimming on sole thickness in pasture-based dairy cattle. Therefore, the principal aim of this study was to evaluate the effect, over a lactation, of trimming on sole thickness, estimated by measuring, using ultrasound, the distance from the external claw sole surface to the tip of the distal phalanx (DDP). A secondary objective was to observe the effect of DDP at drying off on the hazard of increased locomotion scores, i.e., locomotion scores ≥1 (imperfect gait scores) and ≥2 (clinical lameness) in the subsequent lactation.

## 2. Materials and Methods 

All the procedures in this study were approved by Massey University Animal Ethics Committee (protocol number 8/22).

### 2.1. Animals and Farm Location 

The study was undertaken with a 940-cow spring-calving dairy herd in the Manawatu-Wanganui region of New Zealand between May 2018 and May 2019. Most of the cows were Friesian and Jersey crossbreeds, with roughly 10% Friesian cows. Animal age ranged from 2 to 10 years (with a mean age of 4 years). The milking cows were managed as two equal-sized groups grazed separately on the same farm, rotating around the farm’s paddocks. No housing was used on this farm; cows remained permanently at pasture during the study period. Both groups were milked twice daily through a 60-unit rotary milking parlor. The average walking distance from grazing to the milking parlor was 2 km; thus, on average, cows walked 8 km/day. On arrival at the milking parlor, the cows were gathered in the collecting yard, which had a concrete floor, until all the cows had arrived (a process that usually took ~30 min) when milking started. As milking through the rotary parlor took an average of 2.5 h, the average cow spent ~0.75 h per milking (1.5 h/day) standing on concrete (in the yard or the parlor).

Regular lame cow management included identification of lame cows during routine operations by farm staff, with veterinary visits to treat lame cows at least every two weeks (more frequently if required). On this farm, staff did not use systematic locomotion scoring before or during the study. Once cows were identified as lame by farm staff, they were put into a lame-cow group until the farmer determined they were no longer lame. This group was kept in a paddock (confined grazing area) close to the milking parlor and milked once daily in the morning. The farm was selected for the study on the basis of the farmer being interested in the study and the close working relationship with the veterinary practice, not on an identified lameness problem or a problem with claw conformation. The leading causes of lameness on the farm were white line disease, sole injury, and foot rot. All lesions were identified using the criteria set out in [31]. Digital dermatitis had not been detected at any time before the commencement or throughout this research.

Visit 1—at dry-off (May 2018). 

At the first trimming visit (May 2018), cattle in the milking herd were randomly assigned to one of two treatment groups (non-trimming (n = 690) and trimming (n = 250)). For this study, at the first trimming visit, 20 cattle in each of the two treatment groups were randomly selected using a random allocation sheet for ultrasound monitoring of DDP. The number of cows per group was based on a number that could be measured over a three-day period without interfering with farm operations or holding cows for too long. A similar number of animals was used in previous research on DDP in pasture-based cattle [13]. The data from [13] suggest that with the repeated measures model, we can detect differences of ~1.0 mm with 18 cows in each group. Cows that had an ultrasound examination were evenly spread through both milking groups and remained in their groups over the whole study period, except for those moved for disease management purposes.

A professional hoof trimmer employed a mobile hydraulic Wopa Pro+ Cattle Crush with accessories to restrain the cows and an angle grinder (a professional trimming disc) and Hauptner hoof trimming knives to trim the claws using the five-step Dutch method described by Toussaint Raven [32]. Trimming was undertaken for three consecutive days at each visit, with ultrasound measurements being undertaken on all three days, with cow numbers being evenly split for trimmed and non-trimmed cows. Briefly, the trimming procedure involved (1) trimming the toe length of the medial claw to 75 mm, leaving 5 to 7 mm thickness in the tip of the toe and sparing the height of the heel; (2) making the lateral claw equal in length and weight-bearing surface to the medial (or as near as possible); (3) making a slope in the soles; (4) reducing the weight in the affected claw (if lesions were present); and (5) removing loose horn and hard ridges.

The hoof trimmer assigned each hind foot to one of four categories: **0,** trim not required; **1,** light trim required (trim to remove the slight difference between the claw height); **2,** medium trim required (trim to correct marked claw height differential); **3,** heavy trim required (trim to correct claw height difference and treat a lesion). Both hind feet were trimmed as per standard practice, and the worst score was recorded as the score for the cow (cows’ trim scores were very similar across both feet).

The ultrasound technique used in the present study was that validated by Laven et al. [17] for the equipment used in this study. Those authors found that caliper-measured sole thickness immediately below the tip of the distal phalanx was less strongly correlated to the ultrasound measurement of sole thickness at that point than to the ultrasound measurement of DDP to the tip of the distal phalanx (0.51 vs. 0.8, respectively) and that the agreement between sole thickness predicted from DDP with caliper sole thickness was better than that between ultrasound sole thickness and caliper sole thickness. Thus, with the equipment used in this study, measuring DDP to estimate sole thickness was easier, quicker, and more accurate than direct ultrasound measurement of sole thickness. This method of DDP as a proxy for sole thickness has been used in the study of the outbreak of claw lesions reported by Mason et al. [15] and in the evaluation of changes in DDP in four herds in New Zealand [13]. In addition, as a previous study by Laven et al. [13] showed that DDP measurement of both hind feet did not provide useful additional information about DDP compared to one hind foot and took twice as long, only the right hind foot was examined using ultrasound. 

For ultrasound monitoring of DDP, cows were restrained in a hoof crush (Wrangler, Whakatane, New Zealand). Once restrained, a cow’s right hind foot was lifted, washed, and dried before measurement. The DDP was then measured at the tip of the distal phalanx of both lateral and medial claws using a portable ultrasound machine (Mindray DP 6600, Mindray, Szechuan, China), as described by Laven et al. [17]. Briefly, a linear probe was inserted into a vinyl glove containing acoustic coupling gel for protection. Then, transmission gel was applied to the claw surface of the sole, followed by placing the probe along the longitudinal claw axis. The probe frequency was set to 5 MHz. Once an appropriate image was obtained, it was frozen, and a measurement from the outer surface of the sole horn to the tip of the distal phalanx was recorded alongside the cow identification number. The cow was then released, with non-trim cows being released directly to pasture and trimming group cows to the head of the trimming queue.

Visits 2 and 3—October 2018 and May 2019. 

The farmer drafted the same individual cows for the ultrasound group (US-trimming and US-non-trimming), and measurements of DDP were made as in visit 1. 

### 2.2. Locomotion Scoring 

Locomotion scoring was performed fortnightly, with cows being scored during the afternoon milking session as they exited the milking parlor using the 4-point (0–3) scale DairyNZ lameness score [33]. The scoring procedure was as described by Werema et al. [34] and was undertaken by the first author, a trained locomotion scorer [34]. Cows were locomotion scored on 19 occasions between 15 August 2018 and 08 May 2019. Data were included for the analysis of locomotion scores only from cows with at least four locomotion scores during that period.

### 2.3. Statistical Analyses 

All data were analyzed using SPSS version 27 (IBM Corporation, Armonk, NY, USA). Descriptive statistics were created for the locomotion scores, claw trimming category, and distance from the external surface of the sole horn to the tip of the distal phalanx data sets. For all models, data are reported using 95% confidence intervals (95% CI) to identify the range of effect sizes (hazard ratio (HR) and mean differences) with which our data are compatible [35].

A multivariable Cox proportional hazards regression was employed to assess the association of DDP at the first visit to the first observed increase in locomotion score from 0 (LS ≥ 1) and to the first observation of clinical lameness (LS ≥ 2). Key predictor variables in both models were the treatment group and DDP of the lateral and medial claws of the right hind foot at visit 1, which were forced into the model, irrespective of their p-value. Age and breed category (see Table 1) and the interactions between DDP and treatment group were also included in the model but were eligible for removal during the backward selection process if *p* > 0.05 and their removal did not change coefficients by >20%. The impact of using both DDPs of the lateral and medial claws in the same model was assessed by measuring the resulting variance inflation factors (VIF) to ensure that there was no consequential multicollinearity. The proportionality assumption was tested using a visual assessment of Kaplan Meier curves and log(−log) plots and, if those were uncertain, by testing the Schoenfeld residuals. Outputs from the models are reported as hazard ratios (HR) with 95% confidence intervals and interpreted as per Gelman and Greenland [35].

Two linear repeated measure mixed models were then created to assess the effect of trimming on DDP over time. The first model only used data from cows in the trimming group. DDP was the outcome variable, cow was the subject variable, visit and claw within cow were the repeated measures, and trim category at visit 1 was the predictor variable. Age and breed were not used in these models as a univariable analysis had shown no association in this dataset between DDP and age or breed category (*p* ≥ 0.6). All possible two-way interactions were added, and backward selection was then used to remove any interactions where *p* > 0.05 (all main effects were kept in the model, irrespective of *p*-value). The second model used data from all ultrasound-monitored cows. Again, DDP was the outcome variable, cow was the subject variable, visit and claw within cow were the repeated measures, and treatment group (trimming vs. non-trimming) was the predictor variable. DDP at visit 1 and calving dates were used as covariates. Again, all possible two-way interactions were added, and backward selection was then used to remove any interactions where *p* > 0.05 (all main effects were kept in the model). The models used were selected on the smallest values of fit statistics for the −2 Restricted log-likelihood. Outputs from the models are reported as mean differences with 95% confidence intervals and interpreted as per Gelman and Greenland [35]. 

## 3. Results

Initially, we aimed to enroll 40 cows, with 20 in each group (claw trim + ultrasound scanning and ultrasound scanning only). Twenty cows were scanned in the non-trimming group, but 18 were scanned in the trimming group (as two cows in that group were accidentally trimmed before scanning). Thus, 38 cows were scanned during visit 1, with a measurement of DDP being achievable in 74/76 claws (as two cows in the non-trimming group each had only one usable image). The age, breed, and calving date of the ultrasound cows in each treatment group are summarized in Table 1.

At visit 2, the farmer presented 32 cows (17 non-trimming and 15 trimming) for examination. Forty-nine usable images were obtained from the 64 claws. Finally, 32 cows (16 from each treatment group) were drafted during visit 3, with 50 usable images being obtained from the 64 claws. Detailed data on usable images acquired by treatment group and visit are presented in Table 2. 

### 3.1. Locomotion Scores and Lameness Prevalence

Data from 35 (trimming group n = 17 and non-trimming group n = 18) cows were included in this analysis as three cows were excluded as they had less than four locomotion scores. The median number of locomotion scores per included cow was 15 and the interquartile range was 5. A total of 16 locomotion scores of 2 (11 cows) were recorded during the study period, with no locomotion scores of 3. The mean prevalence of locomotion scores of 2 across the study period was 3.3%. Only two cows, one from each treatment group, were drafted by the farmer for lameness treatment. Once cows treated for lameness were included, all cows were recorded as having had at least one score of ≥1, and 11 (trimming group n = 5 and non-trimming group n = 6) were recorded as having had at least one score of 2. 

### 3.2. Survival Analyses

The VIF in the regression model for DDP of the lateral claw (DDP-L) and DDP of the medial claw (DDP-M) were 1.9 and 2.1, respectively (indicating a lack of consequential multicollinearity [36]). The initial models thus included breed, age, both DDPs, treatment group, and the two treatment group × DDP interactions. The final models included only the two DDPs and treatment groups. With regard to the effect of treatment group, for the model evaluating the time to LS ≥ 2, our data were compatible with a large range of effects, from a small decrease in hazard through no effect to a large increase in hazard (see HR and 95% CI in Table 3). For time to LS ≥ 1, the effects of our data were compatible with a range of effects, from a large decrease in hazard to no meaningful effect (Table 3). 

Irrespective of the LS target (LS ≥ 2 or LS ≥ 1), neither DDP-L nor DDP-M at first measurement clearly affected the hazard of increased LS. For LS ≥ 1, our data supported a conclusion that there was no meaningful effect as the 95% confidence interval for the hazard ratio (HR) associated with an increase in DDP of 1 mm ranged from a small decrease in hazard to a small increase (see Table 3). However, for LS ≥ 2, our data did not rule out an increase in DDP of 1 mm being associated with a relatively large increase in the hazard of LS ≥ 2 (upper 95% CI 1.77 and 2.53 for DDP-L and DDP-M, respectively).

### 3.3. Effect of Time and Trimming Category at Visit 1

All interactions were removed before the final model was reached. In this model, there was no clear difference between mean DDP-L and DDP-M, with a mean difference of 0.42 mm (95% CI −0.10 to 0.95) (see Table 4a). This was consistent with the finding that in the 103 within-foot claw comparisons, DDP-L was higher than DDP-M on 54 occasions and lower on 49 occasions. In this group of trimmed cows, DDP before first trimming was smaller at visit 1 in May 2018 than in October 2018 or May 2019 (visits 2 and 3, respectively). The mean difference between visits 1 and 2 was 1.97 mm (95% CI 1.26 to 2.67), between visits 1 and 3 was 1.69 mm (95% CI 1.03 to 2.33), and between visits 2 and 3 was 0.28 mm (95% CI −0.54 to 1.10) (see Table 4a). Trim category at visit 1 had a clear effect on DDP, with trim category 2 cows having higher DDP than trim category 1 and 0 cows (mean difference 1.54 mm (95% CI 0.26 to 2.82) and 2.03 mm (95% CI 0.71 to 3.34), respectively (see Table 4a). 

### 3.4. Effect of Time and Treatment Group on DDP

All interactions were removed before the final model was reached. In this model, there was again no clear difference between mean DDP-L and DDP-M (see Table 4b). The covariate (DDP at visit 1) was significant in the linear repeated measure mixed model. For visit 2, an increase of 1 mm in the covariate was associated with an increase of 0.45 mm (95% CI 0.44 to 0.47 mm) in DDP. For visit 3, an increase of 1 mm in the covariate was associated with an increase of 0.45 mm (95% CI 0.43 to 0.47 mm) in DDP. For the trimmed group, mean DDP at visit 1 was 7.38 and 6.87 mm for lateral and medial claws, respectively. The equivalent figures for the non-trimmed cows were 8.06 and 7.88 mm, respectively. Although, in this model, mean DDP-L was higher than DDP-M, there was again no clear difference between the two measures (see Table 4b). Mean DDP at visit 2 in October 2018 was higher than at visit 3 in May 2019, with a mean difference of 0.6 mm (95% CI 0.13 to 1.08 mm) (see Table 4b). Treatment group had no clear effect on DDP, though the trimmed group had a higher mean DDP than the non-trimmed group (mean difference 0.18 mm; 95% CI −0.32 to 0.68 mm). 

## 4. Discussion

The principal aim of this study was to evaluate over a lactation the effect of prophylactic trimming on DDP. The secondary aim of this study was to evaluate the influence of DDP on the interval between calving and increased locomotion score, primarily imperfect gait (LS ≥ 1), but also clinical lameness (LS ≥ 2). 

Our analysis found no clear effect of DDP on either time to LS ≥ 1 or time to LS ≥ 2. However, our data support different conclusions for the two locomotion score targets. For time to LS ≥ 1, our data strongly support no effect, with a narrow 95% confidence interval (see Table 3), limiting the likely true effect of DDP to between a small decrease and a small increase. In contrast, for time to LS ≥ 2, the large confidence intervals mean that we cannot rule out a meaningful association between DDP and time to LS ≥ 2. Our study was limited by the number of cows that showed clinical lameness and by the relatively high mean DDP, which was higher at visits 2 and 3 than the marginal threshold of 8.5 mm suggested by Newsome et al. [12]. We need more data to better characterize the association between DDP and the risk of lameness in pasture-based cows. 

This analysis used locomotion score data from only one trained scorer. Locomotion scoring is subjective in nature, and it does not have 100% specificity or sensitivity [37,38,39]. Thus, it is possible that this scorer could have systematically under- or overscored cows. However, this bias would not have been related to DDP as the scorer did not have access to the DDP results while they were scoring the cattle.

For our principal outcome of DDP change over time and the effect of trimming on that change, we had two models: our first model of the change in DDP over time (which included only cows from the trimming group) evaluated the effect of time (visit 1, 2, or 3) and trim category at visit 1 (no trim required, light trim required, or medium trim required) on DDP. All interactions were removed from the model as *p* ≥ 0.05 so that only the main effects were included. In trimmed cows, DDP increased between visits 1 and 2, with little change between visits 2 and 3 (see Table 4a). It is likely that our estimate of the difference in DDP between visit 1 and subsequent visits is an underestimate as the proportion of claws in which DDP could not be measured was higher at later visits than at visit 1. The most likely reason for being unable to image a claw is that it is thicker than 10 mm [40]; thus, it is likely that the actual mean DDP of the cows at visits 2 and 3 was higher than our estimate. 

Even excluding this potential effect, despite prophylactic trimming being used on the farm for the first time, the DDP was higher at the drying off after trimming than it had been at the drying off before trimming. Interestingly, in this model, the cows with the most horn removed at visit 1 (those cows in trimming category 2) had the greatest DDP at every visit. Thus, trimming category at visit 1 was strongly associated with DDP at visit 1 and, therefore, the visual assessment of the need for trimming by the experienced professional hoof trimmer (using the five-step Dutch method) was closely associated with sole thickness. This meant that it was only cows with thicker than average soles that received a medium trim (extensive paring), while cows with thinner than average soles were determined to need little or no paring. As the conformational changes used to determine the degree of trim were related to DDP, it was very unlikely that the prophylactic trimming used in this study would have resulted in excessively thin soles immediately after trimming.

The second model of the change in DDP over time (which included cows in the trimming and non-trimming groups) evaluated the effect of time (visit 2 or 3) and treatment group (no trimming vs. trimming) on DDP and used DDP at visit 1 as a covariate. Again, no interactions remained in the final model. As in the previous model, the mean DPP at visits 2 and 3 was greater than at visit 1. Including the covariate result, in contrast to the previous model, DDP was clearly lower at visit 3 than at visit 2. The covariate DDP at visit 1 was strongly associated with the DDP at subsequent visits. This finding suggests that DDP is a cow-related effect (i.e., even when mean DDP changes with time, cows with higher than average DDP will still have higher than average DDP at subsequent time points). We found no effect of treatment on the relationship of DDP with time. In particular, this study suggests that it is very unlikely that the trimming procedure employed in this study would result in a clinically important decrease in DDP (lowest 95% CI −0.32 mm, i.e., our data suggest that it is unlikely that the true effect would be to reduce DDP by more than 0.32 mm). Thus, on this farm, after the sole horn was removed by trimming, the claws were able to respond by increasing horn growth and restoring most or all of the lost horn [41].

Thus, the changes in DDP over time (in particular, the difference in DDP between visits 1 and 3, which were conducted at the same time in the lactation cycle) were not the result of trimming but of differences in hoof growth and wear between years. The published literature on DDP in pasture-based cattle is limited. As far as the authors are aware, the only peer-reviewed published study is that by Laven et al. [13]. This study involved recording the DDP of 25 heifers on five occasions during their first lactation, between days 10 and 220 days post-calving. The results reported by Laven et al. [13] are not the same as ours. Firstly, they found an effect of the claw on DDP, whereas we did not. Secondly, in their study, they reported that mean DDP decreased from day 10 to day 100, whereas our study reports that DDP increased between visit 1 (prior to calving) and visit 2 (mean time since calving ~50 days). These differences may partly be due to using a mixed-age population of cows and heifers in the current study (our sample population ranged from heifers that were about to be dried off after their first lactation to multiparous cows being dried off for the eighth time), whereas Laven et al. [13] only used heifers in their first lactation. No effect of age on DDP was found in this study, but this study was not designed to find such an effect, with age being recorded as a potential confounder rather than a predictor variable of interest). This argument also applies to another potential source of difference: breed. Laven et al. [13] used more Friesian heifers in their study, whereas the large majority of cattle in this study were Friesian × Jersey (see Table 1). No effect of breed was found in this analysis, but the study was not designed to find such an effect. However, the difference between the current study and that by Laven et al. [13] in the change in DDP in early lactation may simply be a herd effect. Laven et al. [42] reported the results of the DDP examination of heifers in four herds and stated that DDP increased between days 0 and 50 in one of the four herds. More data on DDP over time in pasture-based herds are required. 

## 5. Conclusions

The results of the present study suggest that in pasture-based dairy cows, DDP at drying off has no effect on time to LS ≥ 1 in subsequent lactations but that more data are required before conclusions can be drawn on the association between DDP and time to LS ≥ 2 (i.e., clinical lameness). On this farm, DDP increased after calving and it was not affected by trimming. This may be related to our finding that in cows that were trimmed, the degree of trimming required at drying off was strongly associated with DDP in subsequent lactations. This study has demonstrated that if hoof trimming is done correctly in pasture-based cattle, it does not result in decreasing sole thickness over the medium-to-long term. Further studies are warranted to determine the influence of prophylactic hoof trimming on DDP in pasture-based cattle.

## Figures and Tables

**Table 1:** Comparison of age, breed, and calving date between trimming and non-trimming groups (n = 38).

Treatment Group	Age—Number of Cows (%)			Breed—Number of Cows (%)		Calving Date	
	≤4 Years	5–7 Years	≥8 Years	Friesian	F × J	Mean (95% CI)	Median
Non-trimmed	9 (45)	10 (50)	1 (5)	3 (15)	17 (85)	24/08/18 (14/08–03/09)	21 August 2018
Trimmed	8 (44)	9 (50)	1 (6)	2 (11)	16 (89)	17/08/18 (10–23/08)	14 August 2018

F = Friesian, J = Jersey, % = percentage, CI = confidence interval, Min = minimum, Max = maximum.

**Table 2:** Summary of the data on the usable images obtained by treatment group and visit.

Visit	Group	Cows Scanned	Number of Usable Images per Cow	Cows
1	Trim	18	0	0
			1	0
			2	18
	Non-trim	20	0	0
			1	2
			2	18
2	Trim	15	0	0
			1	4
			2	11
	Non-trim	17	0	3
			1	5
			2	9
3	Trim	16	0	3
			1	0
			2	13
	Non-trim	16	0	3
			1	2
			2	11

**Table 3:** The coefficients of the predictor variables on the hazard of LS ≥ 1 and LS ≥ 2 (from the Cox proportional hazards model).

For LS ≥ 2	HR	95% CI	
DDP-L	1.05	0.62	1.77
DDP-M	1.33	0.70	2.53
Treatment group (trim vs. non-trim)	1.34	0.35	5.15
For LS ≥1			
DDP-L	0.93	0.72	1.21
DDP-M	0.95	0.70	1.28
Treatment group (trim vs. non-trim)	0.45	0.20	1.03

LS = locomotion score, CI = confidence interval, DDP-L = distance to the distal phalanx lateral claw, measured during visit 1 (May 2018), DDP-M = distance to the distal phalanx medial claw, HR = hazard ratio (for DDP, this is the increase in hazard given a 1 mm increase in DDP).

**Table 4:** Effect of claw trimming, claw, visit, trim category, and treatment group on mean distance (mm) measured from the external sole surface to the distal phalanx on one dairy farm during the 2018/2019 production season.

a. An evaluation of the association between trim category and DDP (n = 18)					
				95% Confidence Interval	
Claw	Visit	Trim Category at First Trimming	Estimated Marginal Mean	Lower Bound	Upper Bound
Lateral			8.98	8.44	9.52
Medial			8.56	8.02	9.09
	1		7.55	7.05	8.06
	2		9.52	8.82	10.22
	3		9.24	8.55	9.92
		0	7.93	7.48	8.39
		1	8.42	8.05	8.78
		2	9.96	8.71	11.20
**b. An assessment of the effect of prophylactic claw trimming on DDP over time (n = 38)**					
				95% Confidence Interval	
**Claw**	**Visit**	**Treatment Group**	**Estimated Marginal Mean**	**Lower Bound**	**Upper Bound**
Lateral			9.07	8.73	9.40
Medial			8.96	8.63	9.29
	2		9.31	8.95	9.68
	3		8.71	8.40	9.02
		0	8.92	8.56	9.28
		1	9.10	8.77	9.43

Visit: the first trimming was conducted during dry-off (May 2018), the second trimming was undertaken in early lactation (October 2018), and the third trimming was performed at the end of the lactation (May 2019). Trim category: 0 = no trim required, 1 = light trim required, 2 = medium trim required. Treatment group: 0 = non-trimmed cows, 1 = trimmed cows.

## Data Availability

Data are available upon request from the corresponding author.
